# Supplementary material for: Scrambling the skin: A psychophysical study of adaptation to scrambled tactile apparent motion
Source: PLoS One. 2020 Dec 31;15(12):e0227462. doi: 10.1371/journal.pone.0227462 (PMC7775071; doi:10.1371/journal.pone.0227462)
Supplement: Attachment — Submitted filename: Response to Reviewers.docx [file pone.0227462.s001.docx]

*Our replies are in blue italic.*

1. Is the manuscript technically sound, and do the data support the conclusions?

The manuscript must describe a technically sound piece of scientific research with data that supports the conclusions. Experiments must have been conducted rigorously, with appropriate controls, replication, and sample sizes. The conclusions must be drawn appropriately based on the data presented.

Reviewer #1: Partly

Reviewer #2: Partly

2. Has the statistical analysis been performed appropriately and rigorously?

Reviewer #1: No

Reviewer #2: Yes

3. Have the authors made all data underlying the findings in their manuscript fully available?

The [PLOS Data policy](https://aus01.safelinks.protection.outlook.com/?url=https%3A%2F%2Fprotect-au.mimecast.com%2Fs%2FxJ23C5QZ29FZZwZnVFxBHsm%3Fdomain%3Dplosone.org&data=02%7C01%7Cjanet.taylor%40ecu.edu.au%7C00252db3deb442e541df08d7b57ed5a2%7C9bcb323d7fa345e7a36f6d9cfdbcc272%7C1%7C1%7C637177428667633373&sdata=fS31X8qVm9pxW5I8%2FTXu9lYuBdFJLmyxJ0jI8BgOFNI%3D&reserved=0) requires authors to make all data underlying the findings described in their manuscript fully available without restriction, with rare exception (please refer to the Data Availability Statement in the manuscript PDF file). The data should be provided as part of the manuscript or its supporting information, or deposited to a public repository. For example, in addition to summary statistics, the data points behind means, medians and variance measures should be available. If there are restrictions on publicly sharing data—e.g. participant privacy or use of data from a third party—those must be specified.

Reviewer #1: Yes

Reviewer #2: Yes

4. Is the manuscript presented in an intelligible fashion and written in standard English?

PLOS ONE does not copyedit accepted manuscripts, so the language in submitted articles must be clear, correct, and unambiguous. Any typographical or grammatical errors should be corrected at revision, so please note any specific errors here.

Reviewer #1: No

*The reviewer made no further comments to explain or illustrate this point, and we could not identify faults with intelligibility of the paper or our English. We could therefore not address this concern.*

Reviewer #2: Yes

5. Review Comments to the Author

Reviewer #1: The authors reported a variant of motion after effect using six vibrotactile devices. They conducted psychophysics experiment examining the effect of two different adapting stimuli. One adapting stimulus is called as “orderly” condition, in which the adapting vibrotactile feedback is provided in a one-way direction. Another adapting stimulus is called as “scrambled” condition, in which the adapting vibrotactile feedback is presented in a spatially zig-zag fashion. Authors compared the d-prime of these two conditions with non-conditioning situation, and found that d’ was smaller when the adapting stimulus was “scrambled” rather than “orderly” adapting stimulus. The authors also reported the subjective feelings of adapting stimulus.

The authors reported an intriguing phenomenon, however, it was too early to draw a conclusion from the current result. For example, the authors started that the “scrambled” condition is as if it were simulating skin re-arrangement in the central nervous system. It is quite hard to generalize that the somatosensory mapping is corrected based on current psychophysical experiment. If the authors would argue the possibility, the authors should have conducted a series of psychophysical experiments before drawing the conclusion.

Especially, this reviewer would like to clarify the following items before accepting this manuscript.

(1) Without examining neural responses, the authors discuss one possible mechanisms of organization of sensory maps. Why could the authors lead to a discussion on the mechanisms of organization of sensory maps only from one psychophysics experiment?

*It is not necessary to examine neural responses or conduct any particular number of experiments in order to make a hypothesis/discuss possible sensory mechanisms (a famous historical example is Young-Helmholtz theory of colour vision from the first half of the 19^th^ century, which proposed a very specific neural mechanism many years before it was possible to test it; for more examples, see Read JCA.* [*The place of human psychophysics in modern neuroscience*](http://dx.doi.org/10.1016/j.neuroscience.2014.05.036)*. Neuroscience. 2015; 296:116–29.).*

*This study was designed to test a particular hypothesis that is based on published literature, which we cited in the Introduction. The result provides evidence that is consistent with the hypothesis. Note that the paper does not conclude that the hypothesis is correct but rather that the findings are consistent with the hypothesis. This should be clear from the language used in the abstract: “This is the aftereffect we expected…” “We propose that…”, and the conclusion: “an aftereffect consistent with the new arrangement, supporting the idea that motion plays a major role…”*

There was a possibility that the task in the "scrambled" condition was hard to answer for some reasons. Did the authors consider another possibility?

*Yes, we did. Three long paragraphs in Discussion include other possible explanations for the phenomenon and we end with an acknowledgement that these explanations cannot be ruled out (last paragraph of page 18 plus all of page 19; section C). Details are reiterated below.*

*Additional hypotheses were described in detail in Section C of the Discussion. We introduced the section by saying:* “Other types of adaptation may also be relevant in an attempt to account for the effect we observed, and we turn to them now.”; *we explained that frequent direction change might have resulted in weaker motion adaptation in the scrambled condition, and that higher average speed in the scrambled condition might have resulted in stronger adaptation in that condition and that* “it could be partly responsible for poorer direction discrimination in the scrambled condition.” *We conclude section C by saying* “We are currently unable to tease apart possible contributions of different adaptation effects described above.”

(2) Title of current draft was too general. Moreover, “scrambling the skin” is hard to grasp what the authors would like to argue in this manuscript. I would recommend authors to modify the title of this manuscript in a more succinct fashion.

*We prefer to keep this title. We understand that “Scrambling the skin” is a bit unusual but believe it succinctly conveys the essence of our paradigm and is likely to attract interest. The second part of the title, “simulated skin re-arrangement using apparent motion”, refers to the specifics of the method used in the reported study.*

(3) In the abstract, the authors argued that “we propose that the somatosensory system was beginning to ‘correct’…, but how did the authors decide the somatosensory system was to correct…. The somatosensory system may update, or being confused because of unnatural tactile stimulus condition. Why the authors could interpret that the somatosensory system “correct”? Explain.

*Please note that there are nuances in the statement the reviewer has asked about. First, it starts with “We propose…” therefore this is an idea that we are putting forward as a possible explanation rather than something we have decided or concluded. Second, ‘correct’ is presented in quotes. This is because we are using the word in a somewhat unusual circumstance where the somatosensory system is given a deliberately unnatural stimulus. That is, the nervous system is presented with unusual input in which there are repeated reversals of direction, and hence, accelerations, at exactly the same sites on the skin in the middle of otherwise smooth motion. We propose that because this type of motion on the skin is extremely unlikely to be caused by real-life object motions, the somatosensory system is driven to update itself to eradicate these accelerations. That is, the unnatural tactile stimulus is taken to indicate an error in the somatosensory system and the system ‘updates’ or ‘corrects’ itself so that the motion is perceived as smooth.*

*In the original version of paper, we quoted other authors who suggested a similar self-correcting process* *(New and Scholl, reference 30, proposed one to account for motion-induced blindness (MIB):* “In MIB, a small object always falling on the same segment of the retina amidst a dynamic visual field quickly fades from awareness.”).
—
I would argue to accept this manuscript before the items mentioned above is going to be accepted.

Minor points:
(4) The authors should have written the version of SPSS.

*We added the version of SPSS (p. 11).*

(5) How the authors determine the number of participants, and what was the effect size?

*NUMBER OF PARTICIPANTS: This question seems to be about power. We did not conduct an a priori power analysis because the effort would likely be a (complicated) exercise in very poorly informed estimation (we would have to assume variances of residuals and random effects, among other values; see Brysbaert & Stevens, 2018 and Westfall et al, 2014). Apart from the number of participants (we had 20), number of stimuli per participant (we had 648 per participant, or 288 after correction for baseline), study design and analysis all influence the power of a study. We used within-participant (repeated), fully crossed experimental design, linear mixed modelling to account for random effects, and we applied Bonferroni correction when testing contrasts. All the models we tested in the LMM analysis have consistently shown a significant effect of Motion condition.*

*EFFECT SIZE: The effect size we reported as a primary outcome measure is d’ (d-prime), based on the Signal Detection Theory (SDT). It indicates sensitivity for motion direction, computed separately for each individual participant at each ISOI using their baseline-corrected responses in Scrambled and Orderly conditions; separate d-primes were computed at each level of control variables, resulting in 24 separate d-primes per participant, or 4 per experimental condition (3 ISOIs x 2 Motion conditions). The estimated marginal means for 20 participants are reported in Table 2, Fig. 5, and in text on p. 12. The mean effect of Motion Condition was 0.31, close to the typical effect size obtained in psychology of 0.4 (Kuhberget al, 2014).*

*If the reviewer queried about the standardized effect size, such as Cohen’s d, we note that d’ from signal detection is essentially the same concept except for the fact that proportions of hits and false alarms are used to estimate the distance between the means (see p. 24, Herzog et al, 2019). Nevertheless, we can also compute standardized effect size for the mean difference(s) between our conditions. Given that our mean differences are already expressed as d’, standardized effect size should be similar to them. Our computations detailed below confirm this expectation, although standardized sizes were somewhat smaller.*

*Computation of standardized effect size from mixed models involves a complicated denominator – pooled variance components from the model (see equation on p. 2023 in Westfall et al, 2014). In our study, the variance components were random intercept, random slope and separate residual variances for each combination of Motion condition and ISOI. We computed standardized effect sizes of 0.16, 0.27 and 0.16, respectively, for our mean differences (expressed as d-primes) of 0.22, 0.31 and 0.18 (for 75, 120 and 190 ms ISOIs, respectively). Thus, our standardized effect sizes are indeed comparable to, but somewhat smaller, than the effect sizes expressed as d’. Perhaps they are smaller because the residual variance in the denominator was overestimated (pooled residual variance in the above equation does not take into account the co-variation between residuals, which was non-zero in our unstructured ‘Repeated’ covariance matrix).*

*Studies with greater power give more precise parameter estimates; we were prompted by this question to add the 95% CI to indicate the precision of our parameter estimates (p. 12 and Table 2 of the revised manuscript,).*

Brysbaert & Stevens (2018) [Power Analysis and Effect Size in Mixed Effects Models: A Tutorial](https://www.journalofcognition.org/articles/10.5334/joc.10/). Journal of Cognition, 1(1), 9.

Herzog M, Francis G & Clarke A (2019) [Understanding statistics and Experimental Design](https://link.springer.com/book/10.1007%2F978-3-030-03499-3), Springer.

Kuhberger A, Fritz A, Scherndl T (2014) [Publication Bias in Psychology: A Diagnosis Based on the Correlation between Effect Size and Sample Size](https://journals.plos.org/plosone/article?id=10.1371/journal.pone.0105825). PLoSONE 9(9): e105825.

Westfall J, Kenny D, Judd C. [Statistical Power and Optimal Design in Experiments in Which Samples of Participants Participants Respond to Samples of Stimuli](https://www.journalofcognition.org/articles/10.5334/joc.10/). J Exp Psychol Gen. 2014;143(5):2020–45.

(6) The authors excluded 30 responses because the authors judged these 30 data points were outliers. How did the authors determine the criteria of outlier? Explain.

*We considered d’ and c calculated from 30 responses in 120 ms Baseline condition in one individual as outliers because the d’ and c were both beyond 2.5 SD from the respective means for the given condition (as now explained in the first paragraph, p. 11).*

Reviewer #2: A central problem in neuroscience is how the spatial organization of receptors in the periphery is mapped into central neurons in the sensory cortex, and how this mapping is shaped by stimuli. In this study, the authors evaluated a century-old hypothesis that motion across the skin is important for the organization of spatial maps in the brain. To test this hypothesis, they run and experiment were participants were requested to discriminate the direction of apparent motion produced by the sequential activation of vibrating tactors. Before each test stimulus, a priming stimulus was delivered were the activation of two neighbours tactors was occasionally inverted (Scrambled) or not (Orderly). The Scrambled priming was associated with a reduced motion sensitivity. However, this did not produce a systematic bias in motion direction that would provide a stronger support to their hypothesis.

The experimental question is interesting. In general, the study is technically sound. I have major concerns on the type of stimuli used and on the very short duration of the priming stimuli. See below for detailed comments.

In the classical study by Merzenich and Jenkins, plastic changes in the cortical organization followed several months after the skin flap surgery. In (Rosselet et al 2008), the animal was stimulated for 7 h with a brushing stimulus. Instead, in the current study, the duration of each priming stimulus last only 66 seconds, and Scrambled stimuli were immediately followed by test stimuli were tactors were activated in a sequential order. It seems extremely unlikely that such short stimulus may produce a reorganization in the somatosensory cortex…

*We tested the subtle perceptual changes one would expect to occur, and gradually increase in strength, while new synaptic relationships are being established – which could then be measured at the cortical level. We made a statement to that effect on p. 18 (”Our dependent measure was perceptual response, and the proposed (beginning of) neural map change is hypothetical.”), and cited some relevant work.*

*We now cite more studies that show experience-induced rapid changes in brain activity (p. 18, paragraph 1), and emphasize that “*We do not suggest that all rapid, adaptation-type changes are potential precursor to more permanent map changes, but that some are.” (page 18, paragraph 2). *We also add that:* “Consistent changes across a number of spatial tasks would be strong evidence of structural changes, as distinguished from stimulus-specific unmasking of long-range and feedback connections*.*” (page 20, paragraph 3).

*(Please note also that the total (cumulative) duration of conditioning we delivered in each session was 66 s x 24 repeats, i.e., approximately 26 min.)*

The second issue is the usage of apparent motion by sequential activation of vibrotactor. The simplest explanation is that participants might have perceived the Scrambled priming for what it is, i.e., a random activation of tactors, rather than adjust their spatial map according to the novel stimulus.

*Sequential activation gives rise to apparent motion in touch as it does in vision, and disruption of the sequence in the Scrambled stimulus was in the middle of the motion path, after motion event was established. According to our questionnaire, participants did not perceive the Scrambled stimulus as random activation of tactors; it was almost indistinguishable from the Orderly (see Fig. 7).* As described on p. 14 (paragraph 1 of Phenomenological reports), “all participants in both conditions felt motion up and down the forearm, mostly along the straight line, with occasional irregularities in the motion path (gaps, curves, zig-zag motion, twists and turns).” *The smoothing of the Scrambled stimulus in perception is consistent with failure to perceive transient changes in visual motion (we cited the relevant studies in the second paragraph of the Discussion in the original manuscript).*

Altogether, the short duration of the priming stimulus and the use of apparent motion may explain why the authors did not find a reversal in motion perception, but only a reduced sensitivity. Instead, the conclusion that “the somatosensory system was beginning to correct reversed local motion” is not sufficiently supported by the data.

*We agree with the reviewer that much longer duration intervention is likely to be necessary for full reversal of motion and this idea is put forward in the abstract and discussion in several places. In the abstract, we said “*its maximal expression would be a complete reversal in perceived motion direction between locations 3 and 4 for either motion direction”; *to which we now add “*which we expect would occur following much longer conditioning”.

*Nonetheless, subjects were less able to identify the direction of the test stimuli after the scrambled stimulus than the orderly stimulus and this is clear evidence that the two stimuli resulted in different effects on the nervous system. Moreover, the direction of effects is consistent with the hypothesis that the experiment was designed to test. We note that the reviewer does not offer a different explanation for the result that is better supported by the data.*

Minor issues

The authors used signal detection theory to analyse the responses in the discrimination task. This is the “standard” method for detection tasks. It is also correct using it for discrimination tasks; however, using a psychometric function is a more common choice in this second case (e.g., probability of perceiving proximal motion w.r.t. motion direction). The discriminability of the stimulus and motion bias could be analysed in term of JND and PSE, respectively. The analysis can be extended to the sample population by means of General Linear Mixed Model - see for example (Agresti, 2002; Bates, Mächler, Bolker, & Walker, 2015). In case of two levels of the predictor (proximal and distal direction) GLMM are also called “models for matched pairs”.

*Signal detection theory is also a standard method for discrimination tasks (also called recognition tasks – MacMillan & Creelman, 2005, Detection theory: A User’s Guide). We quote from p. 1: “If neither stimulus is null, the experiment is called recognition (…). The methods for analysing detection and recognition are the same, and we make no distinction between them”.*

*The alternative proposed by the reviewer, psychometric curve, would have both motion direction and the ISOI represented on the x axis, ranging from -190 ms (negative sign representing motion in one direction) to + 190 ms (positive sign, opposite direction). JND would indeed inform about sensitivity, and PSE (here, the point of subjective stationarity), about bias. The problem with this approach is that it would not allow the 120 ms ISOI to be ‘special’ as our analysis did. Since our conditioning stimulus had ISOI of 120 ms, it was possible that any aftereffect would be strongest for that velocity (notwithstanding a lack of evidence for speed channels in touch, as reported in our previous psychophysical study - McIntyre et al, 2016,* [*http://jn.physiology.org/lookup/doi/10.1152/jn.00460.2015*](http://jn.physiology.org/lookup/doi/10.1152/jn.00460.2015)*). The 120-ms test indeed had the largest effect size (although we did not find a significant interaction between Motion condition and ISOI).*

*It is also not clear what advantages the proposed alternative analysis would have. The reviewer did not suggest any, or find fault with our analysis.*

It is not clear why the authors pre-processed the data in R and used SPSS for statistical inference. R is an excellent language for model fitting! See for example (Knoblauch & Maloney, 2012) for fitting psychophysical data in R, including GLMM. (However: I am fine with SPSS if the authors prefer using it).

*To satisfy reviewer’s curiosity, data pre-processing was done by one person, and analysis by another.*

6. PLOS authors have the option to publish the peer review history of their article ([what does this mean?](https://aus01.safelinks.protection.outlook.com/?url=https%3A%2F%2Fprotect-au.mimecast.com%2Fs%2F3-OTC6X13RtrrZr3nHLWEee%3Fdomain%3Djournals.plos.org&data=02%7C01%7Cjanet.taylor%40ecu.edu.au%7C00252db3deb442e541df08d7b57ed5a2%7C9bcb323d7fa345e7a36f6d9cfdbcc272%7C1%7C0%7C637177428667643370&sdata=1ef6Rxh6wTOALX3l1qxyZwdYIJPprf2sPgAGn9Dwcj8%3D&reserved=0)). If published, this will include your full peer review and any attached files.


If you choose “no”, your identity will remain anonymous but your review may still be made public.

**Do you want your identity to be public for this peer review?** For information about this choice, including consent withdrawal, please see our [Privacy Policy](https://aus01.safelinks.protection.outlook.com/?url=https%3A%2F%2Fprotect-au.mimecast.com%2Fs%2F0Q4IC71ZgLtAAnAPOC03Du7%3Fdomain%3Dplos.org&data=02%7C01%7Cjanet.taylor%40ecu.edu.au%7C00252db3deb442e541df08d7b57ed5a2%7C9bcb323d7fa345e7a36f6d9cfdbcc272%7C1%7C1%7C637177428667643370&sdata=3y1l%2FBeruRfgAeFTY3A8sMZ5PVIFYRxffEa7qS7KmNU%3D&reserved=0).

Reviewer #1: No

Reviewer #2: No


[NOTE: If reviewer comments were submitted as an attachment file, they will be attached to this email and accessible via the submission site. Please log into your account, locate the manuscript record, and check for the action link "View Attachments". If this link does not appear, there are no attachment files to be viewed.]


While revising your submission, please upload your figure files to the Preflight Analysis and Conversion Engine (PACE) digital diagnostic tool, [https://pacev2.apexcovantage.com/](https://aus01.safelinks.protection.outlook.com/?url=https%3A%2F%2Fprotect-au.mimecast.com%2Fs%2FXiwIC81Zj6t66B6J7sRDmg1%3Fdomain%3Dpacev2.apexcovantage.com&data=02%7C01%7Cjanet.taylor%40ecu.edu.au%7C00252db3deb442e541df08d7b57ed5a2%7C9bcb323d7fa345e7a36f6d9cfdbcc272%7C1%7C1%7C637177428667653363&sdata=l8iDfZF3KMUifW20opg7zO1HLxZtZhliE9K55gd1fIA%3D&reserved=0). PACE helps ensure that figures meet PLOS requirements. To use PACE, you must first register as a user. Registration is free. Then, login and navigate to the UPLOAD tab, where you will find detailed instructions on how to use the tool. If you encounter any issues or have any questions when using PACE, please email us at [figures@plos.org](mailto:figures@plos.org). Please note that Supporting Information files do not need this step.
