# Supplementary material for: Scrambling the skin: A psychophysical study of adaptation to scrambled tactile apparent motion
Source: PLoS One. 2020 Dec 31;15(12):e0227462. doi: 10.1371/journal.pone.0227462 (PMC7775071; doi:10.1371/journal.pone.0227462)
Supplement: Attachment — Submitted filename: Reply to reviewers 2nd round.docx [file pone.0227462.s002.docx]

**Common reply to Reviewers 1-3**

There was a common theme in all reviews, described by the third reviewer as “serious reservations (…) about the framing and interpretation” of the results (underline added). We will briefly outline our response here and give more specific replies to each reviewer below.

Firstly, we note that the reviewers don’t object

1. to the logic of the hypothesis presented in the introduction about the potential role of motion in creation of maps
2. to the scrambling paradigm we developed, *except* for the duration of the conditioning stimulus.

They object to its short duration because

3. we interpret the findings in relation to the hypothesis about plasticity in somatosensory maps, which everyone (including us) agrees would require long-term changes in the statistics of input; this criticism is condensed in the statement that “If the authors wish to talk about what happens when adapting long timescales, they must test longer timescales.” (Reviewer 3)

**Our response**:

Short conditioning produced results *consistent with* the proposed role of motion: degraded direction discrimination may well be a stage on the path to a total reversal in perceived direction. (We added text on pp. 6-7, Introduction, to explain this more clearly). We did not say that our results proved we were on that path. We intend to use long-lasting stimuli and are currently building a wearable device to allow it. Our research capacity is limited and we cannot easily run more experiments as suggested by one reviewer. Since science is a collective and cumulative process, withholding information about the current findings, or framing them in a way that obscures one novel and theoretically well-founded paradigm until we have answers to further questions seems to us not only unnecessary but counterproductive. We do not wish to change the current framing for the study because it is forward looking and because others can test our predictions.

One should always consider alternative explanations, which we have done in the original discussion. Perhaps we did not convey clearly enough that the speed-adaptation explanation is not straightforward. We expanded text on pp. 20-21 to make this clearer.

In brief, higher speed only occurred between pairs 2 and 4, and 3 and 5 (*not* between the test pair, 3 and 4), implicating motion neurons with receptive fields larger than the span from 3 to 4. Many direction-selective neurons increase firing with speed, and should adapt to both speed and direction of motion. Importantly, those adaptations would tilt the balance in opposite directions and it is not clear which would win: the speed-induced adaptation in the scrambled pattern, or direction-induced adaptation in the orderly pattern. Prior psychophysical speed-adaptation studies [26, 27] are silent on these questions, and there is not much other psychophysical research on the topic.

Notwithstanding what we said above, we also don’t want to mislead our readers, and the consistent reaction of all reviewers (although Reviewer 1 seemed satisfied with our initial response) indicates that the emphasis on the map change in most visible parts of the paper (title, abstract, conclusion) was potentially misleading. We changed those parts of the text to more clearly convey (a) the value we place on the motion scrambling paradigm itself, (b) the ambiguity of the present findings, and (c) suggestions for further tests of our hypothesis. We also emphasized the continuity between proposed short-term and long-term changes in the Introduction (pp. 6-7) and Discussion (3^rd^ paragraph on p. 19), and compressed parts of the Discussion to compensate for the additional text.

Separate replies

Reviewer #1: I appreciated that the authors clarified the items that I had raised in the previous review process. I'm now getting comfortable with the authors' responses, but still, I would like to point out the following items.

(1) Thank you for the authors' efforts on additional statistical descriptions showing the effect size (such as confidence interval). I had looked at the data that the authors uploaded in the data repository (in SPSS format), and conducted some trials and errors before adopting the reported model as indicated in Table 2. The readers would benefit from the fact how the authors had chosen a quadratic model for the difference in sensitivity (d') between orderly and scrambled conditions from baseline, and a linear model for the difference in absolute bias.

Also the authors had decided which model could describe the data the best based on information criteria, so it would be helpful for readers to conduct the model selection process in the data analysis section.

*We are glad that the Reviewer chose the same model that we have. We added a paragraph to the Data analysis section (p. 13) to explain further how we choose models and decide whether to include quadratic terms. In short, we usually test for a quadratic term of a continuous predictor variable, but if it is not of central interest and does not improve the model, we don’t include it in the final model.*

Minor comments:

(2) I'm still not very comfortable with the title. Even I would admit the catchy running title ("Scrambling the skin"), there is room to improve in the second part of the title. I initially imagined that the authors conducted a computational simulation of neural representation when I glanced at the title "simulated skin re-arrangement". Moreover, some of the readers may misinterpret "using apparent motion" as "using (visual) apparent motion", even though the authors addressed a question using tactile apparent motion. This is my personal opinion though.

*We changed the second part of the title to make it clear that scrambling is a research paradigm for psychophysical studies:* Motion-scrambling paradigm for psychophysical study of somatosensory plasticity’

(3) I did not notice in the first review process, however, I realized that the degrees of freedom of numerator and denominator in F distribution were not correctly indicated (p.12, F(78.2, 1) = 7.82 should be F(1, 78.2), and other similar four mistakes in the same paragraph).

*Thank you – corrected.*

*****

Reviewer #2: In this study, the authors evaluated a century-old hypothesis that motion across the skin is important for the organization of spatial maps in the brain.

In the first review, both me and the other reviewer expressed major concerns about the experimental design, the analysis, and the conclusions of the study. In particular, I raised two major issues:

1. The very short duration of the conditioning stimuli (here the total duration of conditioning stimuli across the experiment is irrelevant because conditioning stimuli are alternated with "non-scrambled" control stimuli, restoring the putative mapping)

*We addressed the question about duration to some extent in the first round, and more so now. Please see Common reply. Also note that in each session either only scrambled sweeps or only orderly sweeps were presented. The test stimuli presented between conditioning stimuli only involved locations 3 and 4. Without the context of locations 1,2,5 and 6, the test stimuli (3-4 or 4-3) are neither scrambled nor orderly and are not expected to influence (restore or otherwise) mapping.*

2. The usage of apparent "scrambled" motion instead of "scrambled" slip motion. Apparent motion does not reproduce important features of tactile motion, such as shear force and skin strain propagation.

*This is a new comment (slip motion was not mentioned in the original review; the Reviewer then proposed that apparent motion results in percept of “random activation of tactors”, without making it clear that his/her concern was the absence of shear force in particular), and we very much agree that shear forces matter. In fact, we were influenced by the excellent review by* [*Essick (1998)*](https://www.sciencedirect.com/science/article/pii/S0166411598800630) *and conducted (to our knowledge) the first study that placed skin stretch and displacement cues in conflict (*[*Seizova-Cajic et al, 2014*](https://link.springer.com/chapter/10.1007/978-3-662-44193-0_53)*). Our apparatus had tactors that tapped the skin and could also pull it one way or the other. We created apparent motion in one direction while skin was stretched in the opposite direction, and this conflict impaired direction discrimination.*

*We also agree that continuous – ‘slip’ - motion could be used in our paradigm. We have added the following sentence on p. 6 to make that clearer: “apparent motion was used in the present study but continuous motion could be used to the same effect, by reversing motion direction along a short segment within a longer trajectory”.*

*However, we do not agree with the view that, because friction clearly matters, we should have used ‘slip’ motion in the present study and not the apparent motion we used. The Reviewer did not explain why this paradigm in particular should require that. If the implication is that all tactile motion studies should preferably use friction/skin strain propagation, again we disagree. Many excellent studies, from those that kickstarted Gestalt perception theory to modern neurophysiological and psychophysical studies, relied on apparent motion – we hardly need to quote them. Rather, we’ll explain why we think it is a valid approach.*

*Firstly, frictionless stimuli, including some ecological stimuli (e.g., gentle air and water currents, crawling insects), result in perception of motion. Second, apparent motion is simply sampled motion, and even ecological stimuli are sampled, both in vision and in touch. The same mechanisms (psychological/gestalt or neural, depending on one’s perspective) that put segmented trajectories of ecological motion together are also activated by experimentally created sampled motion, however artificial our devices are. One could debate sampling density but this does not change the basic argument. Finally, we also believe that, from the methodological point of view, it is justified to isolate one mechanism/cue and study it, before attempting a synthesis.*

I expected major changes in the manuscript; for example, the authors could address the first point with a simple control experiment using the same experimental setup, by exposing participants to a much prolonged conditioning stimulus.

*We have made more changes this time, esp. to emphasize the continuity between proposed short-term and long-term changes, and to make the alternative explanation even more visible (as indicated in Common reply).*

*To explain in some detail why we did not do ‘a simple control’: these experiments are not simple or quick. Our experiment was preceded by extensive piloting, required two experimenters for smooth running, and took three sessions per participant to achieve sufficient experimental control. Participants need to be dedicated and available for even longer time periods for longer conditioning, and this requires funding (we only report results from naïve participants to avoid bias). Slightly longer conditioning could be conducted in the lab using our existing apparatus, but significantly longer conditioning – which we aim for - requires a wearable device to allow the participants to leave the lab. This device is being built at the moment. On that note, one reason that tactile research field is thought to lag far behind vision is the need to build devices, which requires more funding and time than programming of visual stimuli on computer monitors (for more interesting thoughts on the topic, see* [*Hutmacher, 2019*](https://doi.org/10.3389/fpsyg.2019.02246)*). We are shooting ourselves in the foot if we don’t publish available results.*

Instead, the authors only performed minor manuscript changes. The authors added that "Consistent changes across a number of spatial tasks would be strong evidence of structural changes" but they did not test for it.

*Indeed, we made a prediction, which we have not tested yet. Spelling out our predictions aids scientific communication because it brings theoretical clarity and may motivate further study. We now added one new figure (Fig.8) to make the predictions even clearer.*

For the reasons I explained before, I am not convinced that the short stimuli used in the study would produce the relevant plastic changes postulated by the authors.

*Our intent was not to claim that all the expected plastic changes already occurred, but that the observed changes could be the beginning of a more profound change. As indicated in the Common reply, we have made more changes to the manuscript in this revision to make this very clear.*

The electrophysiological studies now included in the manuscript refer to different phenomena, such as the change in the receptive field in cat's visual cortex, and not to the remapping of a skin portion in response to motion.

*Our approach is novel and any studies we cite necessarily refer to phenomena different from remapping of a skin portion in response to motion. The mentioned studies were cited to illustrate that rapid changes in receptive fields may occur following short changes in input. We quote from p. 19:*

“Changes were observed in receptive fields in cats under comparable stimulation regimes within minutes of stimulation…Rapid changes were also shown in S1 activity patterns … suggesting dynamic receptive fields that adjust to specific stimulation patterns.”

The decrease in sensitivity reported by the authors could be due to a simple masking effect; instead, a bias change would provide much stronger evidence for remapping.

*The Reviewer did not explain what masking effect they had in mind, and we are not sure how to interpret this comment. Masking in perception usually refers to the spatial or temporal configuration of stimuli such that one stimulus makes the other (a target) more difficult to perceive. Scrambling and Orderly conditions had the same total amount of stimulation per location; the test stimulus – the ‘target’ - was in both conditions presented on its own a few seconds after the offset of conditioning, and was not followed by another stimulus (not before the participant responded). We don’t think there were conditions for masking, or differences between conditions that could be construed as differences in masking.*

*Similarly for bias change - we are not sure what the Reviewer had in mind. How would bias provide a stronger evidence for remapping than our direction sensitivity task? (We had a bidirectional conditioning stimulus, and the test stimulus in either direction -- we remind future readers of this in paragraph 1, p. 17, Discussion. Different individuals have different biases in choosing proximal vs distal response. We reported both those – signed biases, and absolute bias. The meaning of bias we use comes from signal detection theory and is distinct from sensitivity to direction, which our hypothesis propose should change, eventually reversing. How could bias be a better evidence/test of remapping than perception of direction measured using d’?)*

Overall, I am not convinced that the conclusions of the manuscript adequately supported by the present results.

*As indicated in the Common reply, we have changed the Abstract and Conclusions to make the two interpretations equally visible, and we added Fig. 8 and expanded text with predictions from longer conditioning. We think that our conclusions are consistent with present findings.*

*References*

G.K. Essick, G.K. (1998). Factors affecting direction discrimination of moving tactile stimuli,Editor(s): J.W. Morley, *Advances in Psychology*, North-Holland,Volume 127. <https://www.sciencedirect.com/science/article/pii/S0166411598800630>

Hutmacher, F. (2019). Why Is There So Much More Research on Vision Than on Any Other Sensory Modality? *Frontiers in Psychology*, *10*(2246). <https://doi.org/10.3389/fpsyg.2019.02246>

Seizova-Cajic, T., Karlsson, K., Bergstrom, S., McIntyre, S., & Birznieks, I. (2014). Lateral Skin Stretch Influences Direction Judgments of Motion Across the Skin. In *Haptics: Neuroscience, Devices, Modeling, and Applications* (pp. 425–431). Springer Berlin Heidelberg. <https://link.springer.com/chapter/10.1007/978-3-662-44193-0_53> <https://eurohaptics.org/static/2014/node/39.html>

*****

Reviewer #3: In this manuscript, the authors study the effects of adaptation on motion perception. The authors use two different stimuli, an orderly and a scrambled stimulus, as adaptors, and find that performance is more strongly degraded for the scrambled adaptor. The experiments are well executed and the results and analysis are convincing. However, I share the serious reservations that Reviewers 1 and 2 brought up about the framing and interpretation of these results.

*Please see Common reply. More detailed replies to specific comments are below.*
 
First, the authors claim in their Abstract that their results may represent “one possible mechanism for organization of sensory maps.” In their Conclusion, they claim that their experiments support “the idea that motion plays a major role in organizing spatial maps in touch,” and that “Our simulated-surgery paradigm is a potentially useful tool in the experimental study of plasticity in sensory maps in conscious humans.”

*To address the above quotes one by one: (1) This takes the quote out of context and misrepresents what we said; we were describing a hypothesis and did not say that our results may represent a mechanism; (we said* ” We propose that the somatosensory system was beginning to ‘correct’ reversed local motion to uncurl and remove the U-turns that always occurred in the same location. Such de-correlation between accelerations and their location on the sensory surface is one possible mechanism for organization of sensory maps.”); *nevertheless, we understand that the Reviewer’s objection to all the quotes was to our focus on plasticity, which we address in the Common reply and in more detail below; (2) our conclusion now very explicitly refers to both potential explanations (second paragraph of the conclusion, p. 21) ; (3) we do think that our paradigm is a potentially useful tool, and a sentence to that effect remains in the conclusion (p. 21).*

However, the authors also explain that the results from this study are most likely mediated by short-term adaptation mechanisms, which are almost entirely distinct from the long-term remapping mechanisms that govern the organization of sensory maps. To be more specific, adaptation is a short-term decrease in neural responsivity that occurs in response to an excess of incoming activity, and is thought to depend on gain-control mechanisms internal to individual cortical cells (c.f. Sanchez-Vives et. al. 2000).

*This is indeed a classical view of adaptation. A broader view is well illustrated by the title of Solomon & Koch 2014 paper cited in the original manuscript, also cited by this Reviewer (‘****Moving sensory adaptation beyond suppressive effects in single neurons****’). This review of adaptation studies stresses the importance of surround stimuli i.e., context, in understanding adaptation, and that adaptation does not always result in decreases in activity.*

In contrast, remapping is likely a long-term homeostatic response to a lack of "feedforward" activity, and is associated with the strengthening of existing synaptic connections and growth of new neural connections. Because physiological literature suggests that these are two largely separate processes, it’s not clear to me that the results of adaptation psychophysics can be meaningfully applied to questions of remapping.

*We may have a theoretical disagreement here with the Reviewer, in that we think there is in some instances continuity between immediate perceptual changes (typically labelled ‘adaptation effects’) and potential long-term changes (typically labelled ‘plasticity’). We now added a paragraph on p. 19 about adaptation and plasticity, and we expanded the introduction and discussion to emphasize the gradual changes we expect (as indicated in Common reply). We are not alone in this belief, and we had previously cited other researchers who put forward similar views [refs 32 – 34].*

*In their paper on plasticity vs stability in vision,* *Wandell & Smirnakis (2009)* *also note cases in which it is difficult to draw a line between adaptation and plasticity:*

“In cases in which neural tissue is injured, the two processes typically operate at overlapping timescales in a way that makes them difficult to separate. For example, suppose half of the input signals to a neuron are suddenly silenced. This will alter the statistical structure of the neuron’s input and change its input resistance. Properties such as synaptic gain are likely to change in the short term. In the long term, this may become very difficult or impossible to reverse, and the adaptation may be followed by dendritic sprouting and the formation of new synapses or long-term strengthening of existing synapses, conferring potentially new properties to neuronal circuits” (Box 1)

*Our paradigm and hypothesis are not about the changes triggered by a loss of input. The difference from physical injury is that there is no sudden loss of feedforward activity the Reviewer referred to, but that does not preclude a permanent change; it may simply make it more gradual.*

*We proposed a specific trigger for such a process (on p. 18): “*acceleration needs to consistently occur on the same segment of the sensory surface.”. *We also proposed the stopping rule: decorrelation. This in our opinion comprises a well-rounded and testable theoretical proposal, which extends beyond the historically old idea that motion helps with map formation.*

Second, the authors claim in the Abstract that if subjects were exposed to a much longer duration of adapting stimulation than tested in this study, they would expect “a complete reversal in perceived motion direction.” The data presented in this manuscript cannot distinguish between mechanisms that are weakening the sensitivities at sites 3 and 4 (that is, turning the neural mechanisms off) from mechanisms that swap the sensitivities of sites 3 and 4. I believe that the swapping result would be novel – I am not aware of any prior psychophysical studies in which the ordinal relationship of two spatial locations was reversed due to an extended period of adaptation. Additionally, adaptation effects at different timescales are likely mediated by different mechanisms (c.f. Solomon & Kohn 2014), so results at short timescales may not extrapolate to longer timescales. If the authors wish to talk about what happens when adapting long timescales, they must test longer timescales. As it stands, these data speak to the effects of short-term adaptation.

*These points were addressed in the Common reply. We made changes in several places to make different possible interpretations of the results more visible. One of them is the beginning of map change.*

The authors helpfully point out that the higher average speed of the scrambled stimulus may lead to it being a more effective adaptor than the orderly stimulus. This explanation is well supported by previous work from the corresponding author, and seems to be more parsimonious than remapping.

*As explained in the Common reply, this is not a straightforward explanation because direction adaptation (greater in Orderly) and speed adaptation (greater in Scrambled) give opposite experimental predictions, and we don’t know what the net effect would be. It is thus difficult to say whether this explanation is more parsimonious.*

Wandell, B. A., & Smirnakis, S. M. (2009). Plasticity and stability of visual field maps in adult primary visual cortex. *Nature Reviews Neuroscience*, *10*(12), 873–884. <https://doi.org/10.1038/nrn2741>
