# Supplementary material for: Scrambling the skin: A psychophysical study of adaptation to scrambled tactile apparent motion
Source: PLoS One. 2020 Dec 31;15(12):e0227462. doi: 10.1371/journal.pone.0227462 (PMC7775071; doi:10.1371/journal.pone.0227462)
Supplement: Attachment — Submitted filename: Reply to reviewers and editor submitted.docx [file pone.0227462.s003.docx]

**Reply to the Editor and Reviewers**

**Editor**

*Please keep in mind the comment by reviewer #1, to keep the text simple. As soon as you are having a hard time to explain to tell it in a simple way, it might well be that you are over-interpreting your data.*

We find it unfortunate that the editor supports this reviewer’s request, asking that we simplify the text and proposing that, if it is not simple enough, we are probably overinterpreting. This approach to reviewing manuscripts allows for arbitrary decisions and shows little respect for scientific discussion. We regret that we have to be so blunt but find it important to convey our views and defend authors’ rights. More details are below.

First, the request to ‘keep the text simple’ is not specific; it does not refer to any particular statements we made. We think that the present text is as complicated as it needs to be to convey our thinking about the topic. What makes a text simple or complicated also depends on the reader’s background and we don’t think that the request to simplify the text to satisfy a reviewer who is simply complaining that the text is complex is reasonable.

We think similarly about the suggestion that we might be ‘over-interpreting’. At which point is there ‘too much’ interpreting, and who is to judge? How does one distinguish ‘over-interpreting’ from theorizing? We of course agree that *mis*interpreting must be avoided, and have been happy to address any specific comments about possible misinterpretations.

*Please also follow the suggestions of reviewers #2 and #3 to change the title (#2, #3), i.e., to remove/replace “plasticity”. I encourage you to follow reviewer #3 to include “apparent motion” in the title, because that is how the paradigm is called (and well-known) in vision research.*

We have done so and the new title is: Scrambling the skin: A psychophysical study of adaptation to scrambled apparent motion

We note that our original title had the term ‘apparent motion’ in it (also see our preprint [here](https://www.biorxiv.org/content/10.1101/2019.12.20.884239v1?rss=1)), which one of the reviewers found slightly confusing because to him/her the term implicated vision.

We are removing the term ‘plasticity’ although we are a little surprised by the staunch opposition by all reviewers to its use, as if it repaints everything. As we said in earlier responses to reviewers (also see p. 19 of the manuscript), there is no clear dividing line between adaptation and plasticity (which is why we are happy to use either). A quick search of recent papers presents with an example in which adaptation is described as a ‘rapid form of neural plasticity’ (Akyuz et al, 2020). There seems to be a difference in vocabulary used in different research environments.

Akyuz et al (2020) [Short- and long-term forms of neural adaptation](https://www.sciencedirect.com/science/article/pii/S0010945219304320?casa_token=g6KjTIRGwyMAAAAA:e68r0bYYGF7JOf4-iKvn7mIHxU_27uPAbGW7ubIpsNJ7d-Lcyii-TjIkt-bNRWdjgfmZ6PU5zw): An ERP investigation of dynamic motion aftereffects, Cortex,Volume 125, pp 122-134.

*I also have some comments of my own:*

1. *Abstract: keep to the data and results of the present study, and so please remove the last sentence “Longer conditioning … without reversing.”*

The request to remove this sentence in order to ‘keep to the data’ does not seem appropriate to us. It is a prerogative of authors to theorize. The end of the abstract is a place for a mini-discussion. This sentence explains how the ambiguity in our result can be resolved in the future, and we wish to keep it. We expanded the sentence to make it perfectly clear that we are talking about future research.

2. *Questions remain:*

- *With respect to the baseline, the orderly stimulus is significantly deteriorated. How well is the baseline matched? Possibly better would be a baseline of vibration without motion direction, in order to remove the possible masking effect of stimulating the flanking locations.*

As we explain in the discussion, two types of adaptation lead to deterioration in Orderly relative to Baseline: adaptation to intensity and adaptation to motion. Tests in the baseline were identical to the tests in Orderly and Scrambled conditions and in that sense, they were a perfect match.

Different types of baseline would of course show different things. We chose the baseline that shows the ability to discriminate two motion directions with no prior conditioning. The rationale for the above suggestion is not very clear to us. If the intention is to control for the possible adaptation spread (perhaps this is meant by ‘masking’?) from flanking locations to the middle (tested) locations, note that this control is already incorporated in the Orderly condition. Since our critical comparison was between the Orderly and Scrambled (not Orderly and Baseline), this is sufficient.

*– How well is the “orderly” condition matched to the “scrambled” one? It would be good to have a reference condition that has an amount of “scrambling” in the flanking positions.*

They are extremely well matched; the only difference between them is in the middle two locations, with rationale for this choice explained in detail in the paper. We could have also have used random order of stimulation in the flanking locations, or scrambled pattern, as suggested by the Editor, or some other control stimuli, but the question is what exact purpose would they serve, and how expanding the discussion to list them – as requested by the Editor further down - would improve the manuscript.

*– How is the relation of the distance between the stimulus locations to the receptive field size (e.g. two-point discrimination)?*

Two-point discrimination varies with the exact location on the forearm and is in the order of several centimeters, usually smaller than distance between our vibrators (4 cm). Thresholds depend on the method of delivery (see, e.g., Mancini et al, 2014). Successive stimuli result in lower thresholds than simultaneous stimuli, and successive stimuli that fall in a temporal window that triggers motion can be very close together (below 2-pt threshold) and still be perceived as motion with recognizable direction. Perceived motion smoothness is a function of both distance and temporal parameters (see Kirman paper cited in our manuscript).

Mancini et al (2014). Whole‐body mapping of spatial acuity for pain and touch. Annals of Neurology, 75(6), 917–924. <https://doi.org/10.1002/ana.24179>

*– The explanation heavily focuses on spatial remapping, but an alternative explanation would be temporal remapping.*

Indeed, motion is a spatiotemporal event and an ‘undesirable’ acceleration can be smoothed out by reinterpreting/remapping either space or time. What might change in our paradigm (space or time, or both) and to what extent, is an empirical question and an interesting one, although hard to address. Spatial remapping can be operationalized through individual localization responses (localization of a single point at a time takes the time out of the equation), but there is no easy way to operationalize temporal remapping. One way to do it is via a temporal order judgment (TOJ) task. However, if the TOJ task involves the two locations that might also be spatially remapped, any resulting perceptual bias is ambiguous. To make the temporal task independent of spatial locations, the time judgment should be made relative to an external reference (e.g., a clock). We now briefly mention in the Discussion (p. 19) that temporal order judgment may also change due to motion scrambling, without going into the details laid out above.

*These remaining questions call for further experiments, as you have yourselves agreed. Such experiments need not be included in the present study, but they further stress the preliminary nature of your results.*

*These questions need to be addressed briefly in the discussion, and you need to refrain from over-interpreting your results.*

We already proposed specific experiments to resolve ambiguities identified in interpretation of current results (p. 21, Fig. 8), but the questions about control conditions raised by the Editor are new so we could not have agreed that they require further experiments. We maintain that our control conditions were suitable for the question we asked, and note that the three reviewers did not object to any aspect of study design, except for the duration of conditioning. As we said above, we don’t see that expanding the discussion to list other possible control stimuli would improve it. On the other hand, we think that the possibility of temporal remapping should be mentioned and have added text on p. 19 to address this possibility.

**Reviewer #1**

*Thank you very much for great efforts on revising this manuscript. By this revision, the authors clearly show their attitude that main objective of this study is not to show the psychological phenomenon (degraded motion discrimination after the exposure to scrambled pattern), but to show as an evidence of somatosensory plasticity after conditioning stimulus for 66 seconds (and repeated trials for 24 times).*

We don't understand what the Reviewer is trying to contrast here. We wanted to show the degraded motion discrimination after the exposure to scrambled pattern, this was our prediction. And yes, we are proposing that this is the first stage of a longer process of map change (which can be labelled ‘plasticity’ or ‘adaptation’). The two intentions are perfectly compatible. Possibly the misunderstanding here stems from the meaning the Reviewer attributes to the term ‘plasticity’ as completely incompatible with short-term conditioning (but see our related comment to the Editor when discussing the change of title).

*In addition, the authors updated the abstract and stated that "The 26-minutes conditioning was interspersed with test motion between the two middle tactors presented on their own (sequence 3-4 or 4-3)" even though the actual duration of conditioning stimulus was 66 seconds for each test cycle. This is misleading change and it appears that the authors tried to overstate the observed psychological phenomenon.*

Our original statement was accurate: the conditioning lasted 26 min (more precisely, 26.4 min) and was interspersed with tests, just as we said. We added more details to it (see Abstract), but also wish to make a comment here on the tone of the review: the Reviewer described as ‘misleading’ our accurate description, and has imputed to us an intention to “overstate the observed psychological phenomenon”. The imputations feel unfair and inappropriate. The Reviewer may want to consider how s/he phrases any intended criticisms.

*I fully appreciated that the authors added detail information on statistical analysis procedures and criteria. It helps readers to follow the logic in the data analysis. However, I would like to change my previous decision if the authors would show their data as an evidence of somatosensory plasticity. In current manuscript, the argument is focused on this hypothetical statement, rather not reporting the phenomenon. The text is getting complicated and hard to follow the main psychological result, which was worth publishing. If the authors could modify the manuscript by simply reporting the observed phenomenon, there would be a chance to be published.*

We and the Reviewer have contrasting opinions about what a scientific paper should include. We believe that theory is very important in science and as the current study was conceived on the theoretical basis we propose in the manuscript, and produced the results we predicted, we are primarily interested in reporting the findings in that context, as discussed in previous rounds of reviews and in the above comments to the Editor. Nonetheless, we acknowledge that other explanations are possible for the current results and have done our best to present this in the Discussion.

**Reviewer #2**

*The authors addressed my previous comments. I still have some reservations about the interpretation of the results. I suppose, however, the study can be published, so that the hypothesis can be further tested and refined. I only have two minor comments that can be managed during the editorial process:

I suggest to remove from the title the word “plasticity”, which would be misleading for the readers and replace for e.g. with “re-learning of relative positions” or similar.*

Plasticity is no longer mentioned in the title. *Please expand the Table 1 caption. I am not sure about the meaning of column labels Proximal/Distal (stimulus motion direction?) and Elbow to wrist/Wrist to elbow.*

We expanded the legend and slightly edited the table entries.

**Reviewer #3**

*In my opinion, the new version of the manuscript is much improved. The authors have done a good job of qualifying their original claims of plasticity, and now appropriately consider adaptation as an alternative explanation. I am satisfied outside of a few, relatively minor concerns.

First, I continue to be uncomfortable with the word “Plasticity” in the title of the paper. I understand that the method will eventually be used in a plasticity paradigm, but the data in this paper speak to a shorter timescale process. Any link to plasticity is, at this point, purely speculative. Terms such as “Adaptation” or “Changes in Somatosensory Sensitivity” would be more appropriate.*

Plasticity is no longer mentioned in the title, and adaptation is. *Second, I remain unconvinced by the idea proposed in the Discussion that “frequent direction change” would necessarily lead to weaker motion adaptation than a steady, unchanging motion stimulus. In practice, the short-term adaptation properties (occurring in the first ~100 ms of the response) of neurons in both the somatosensory and visual systems mean that they are often more strongly driven by transient changes in stimulus strength than by steady stimulation (e.g. Connor and Johnson 1990, Lisberger and Movshon 1999). As such, my prediction would run opposite that of the authors, in that I would expect rapidly changing motion stimuli to be a stronger adaptor than consistent motion of the same average speed.
The authors are entitled to their point of view. However, the “frequent direction change” paragraph, as it stands, is mere conjecture. Its claim is much less well supported than the counterbalancing claim that higher speeds lead to stronger adaptation. This point would be strengthened by either references or a more developed argument that supports the idea that rapidly changing motion stimuli are less effective than consistent motion stimuli at driving adaptation.*

It is true that even so called slowly adapting neurons tend to respond with a burst to the onset of an On stimulus. Therefore, the average firing rate could potentially be greater in response to an On-Off-On-Off stimulus, than in response to an On stimulus of the same duration. It may follow that adaptation to the former would be stronger than adaptation to the latter (although not necessarily – we are not aware of a study directly showing that). We understand this to be the gist of the Reviewer’s argument here and it is a fair point. However, there is more: we are talking about motion neurons with large receptive fields (described by Costanzo and Gardner in 1980-ies) that presumably integrate inputs from multiple local motion neurons, as do visual global motion neurons in MT (see Snowden et al, 1994). Those neurons in MT respond more strongly to more coherent motion fields (measured using random-dot kinematograms, RDKs; e.g., Van Wezel & Britten, 2002). Since the Scrambled pattern presents *less* coherent motion within the same time period than Orderly, it is possible that it results in less adaptation (although our linear motion can hardly be compared to RDKs). This is a complicated and speculative argument but we expanded a paragraph on p. 20 to elaborate our point. We also swapped the order of paragraphs containing the two explanations to describe the speed adaptation first.

Costanzo, R. M., & Gardner, E. P. (1980). A quantitative analysis of responses of direction-sensitive neurons in somatosensory cortex of awake monkeys. *Journal of Neurophysiology*, *43*(5), 1319–1341.

Snowden, J., Milne, B., & Santen, V. (1994). Is Global Motion Really Based on Spatial Integration of Local Motion Signals ? *Vision Research*, *34*(Ix), 2425–2430.

Van Wezel, R. J. a, & Britten, K. H. (2002). Motion adaptation in area MT. *Journal of Neurophysiology*, *88*(6), 3469–3476. https://doi.org/10.1152/jn.00276.200

*Please note that the cited text in the replies to reviewers (beginning “In brief, higher speed…”) is not present in the current version of the paper.*

We see now that our formatting in the previous reply to reviewers might have suggested that we included that paragraph in the manuscript. We actually hadn’t intended it to be a part of the manuscript. The Discussion already contains information given there and we were just summarizing it in the reply letter.
